# Prevalence and risk factors for falls in older men and women: The English Longitudinal Study of Ageing

**DOI:** 10.1093/ageing/afw129

**Published:** 2016-11-02

**Authors:** Catharine R. Gale, Cyrus Cooper, Avan Aihie Sayer

**Affiliations:** 1 MRC Lifecourse Epidemiology Unit, University of Southampton, Southampton, UK; 2 Centre for Cognitive Ageing & Cognitive Epidemiology, Department of Psychology, University of Edinburgh, Edinburgh, UK

**Keywords:** older people, falls, prevalence, risk factors

## Abstract

**Background:**

falls are a major cause of disability and death in older people. Women are more likely to fall than men, but little is known about whether risk factors for falls differ between the sexes. We used data from the English Longitudinal Study of Ageing to investigate the prevalence of falls by sex and to examine cross-sectionally sex-specific associations between a range of potential risk factors and likelihood of falling.

**Methods:**

participants were 4,301 men and women aged 60 and over who had taken part in the 2012–13 survey of the English Longitudinal Study of Ageing. They provided information about sociodemographic, lifestyle and behavioural and medical factors, had their physical and cognitive function assessed and responded to a question about whether they had fallen down in the last two years.

**Results:**

in multivariable logistic regression models, severe pain and diagnosis of at least one chronic disease were independently associated with falls in both sexes. Sex-specific risk factors were incontinence (odds ratio (OR), 1.48; 95% CI, 1.19, 1.85) and frailty (OR 1.69, 95% CI 1.06, 2.69) in women, and older age (OR 1.02, 95% CI 1.04, 1.07), high levels of depressive symptoms (OR 1.33, 95% CI 1.05, 1.68), and being unable to perform a standing balance test (OR 3.32, 95% CI 2.09, 5.29) in men.

**Conclusion:**

although we found some homogeneity between the sexes in the risk factors that were associated with falls, the existence of several sex-specific risk factors suggests that gender should be taken into account in designing fall-prevention strategies.

## Introduction

Falls in older people are a major public health issue. They are the most frequent type of accidents in people aged 65 and older, and are the major cause of injury-related hospitalisation in this age group. Injuries caused by falls are associated with disability, loss of independence and increased mortality [1, 2]. The financial costs of falls in terms of use of ambulance services, health and social care are substantial [3, 4]. Even when falls do not result in physical injury, they can cause older people to become fearful of falling, with consequent restrictions on daily activities and onset of functional decline [5, 6].

There is evidence that women have a higher likelihood of falls than men [7–9]. The fact that women experience more loss in bone mineral density than men [10] as a consequence of the menopause may be one explanation for differences in fall and fracture rates. But although many studies have investigated risk factors for falls in older people, very few have included sex-specific analyses and those that did were based on small, unrepresentative samples [11, 12]. Information on current sex-specific fall rates in older people is sparse. One recent large survey of a nationally representative sample in Canada found evidence that associations between a range of potential risk factors and falls differed between the sexes [13]. In order to establish whether gender should be taken into account in the UK fall-prevention programmes, it is essential to establish whether evidence of sex-specific risk factors for falls is present in the UK population-based studies.

We used data from the English Longitudinal Study of Ageing (ELSA) to investigate sex-specific associations between a wide range of potential risk factors and history of falls in the last two years.

## Methods

### Participants

The initial sample for ELSA was based on people aged ≥50 years who had participated in the Health Survey for England in 1998, 1999 or 2001 [14]. It was drawn by postcode sector, stratified by health authority and proportion of households in non-manual socioeconomic groups. The initial survey took place in 2002–3. Subsequent waves of data collection have taken place at two yearly intervals. Refreshment samples drawn from the Health Survey for England were added at Waves 3 and 4 to maintain the representation of people aged 50–75. The current study uses data from Wave 6 (2012–13). Ethical approval was obtained from the NHS Multicentre Research Ethics Committee in London. Participants gave written informed consent.

### Measures

#### Falls

Participants aged 60 or over were asked whether they had fallen down in the last two years (or, in the case of participants who had taken part in the previous wave two years earlier, since the date they were last interviewed) for any reason. There were two responses: yes or no.

#### Independent variables

We selected potential risk factors for falls based on previous evidence [13, 15–22]. Sociodemographic factors included sex, age, marital status (married/has partner, widowed/divorced/separated or single) and socioeconomic position. Socioeconomic position was indexed by total household wealth, including savings and investments, value of any property or business assets, net of debt, excluding pension assets. Lifestyle or behavioural factors included body mass index (BMI), smoking status, physical activity and alcohol intake. Medical factors included a number of prescribed medications, extent of co-morbidity, depressive symptoms, frailty status as defined by the Fried phenotype [23], and problems with frequent pain, incontinence, hearing or eyesight. Physical and cognitive function factors included balance, grip strength, lung function, walking speed and memory. For full details of the assessment of these independent variables, see Supplementary data, available at *Age and Ageing* online.

#### Statistical analysis

Data were weighted to correct for sampling probabilities, non-response and for differential sample loss between Waves 5 and 6. Use of these weights allows correction for non-response at the interview and at the nurse visit. The corrected data should be representative of the English population aged 60 and over. Univariable logistic regression was used to examine the association between each independent variable and the odds of having fallen down in the last two years. Variables that were associated with history of falling with a *P* value <0.2 were included in a multivariable model, as in a previous study [13], having first checked for multicollinearity. In total, 5,879 participants aged ≥60 took part in the interview and nurse visit. The analysis that follows is based on 4,301 participants (73.2%) who had complete data on all variables of interest.

## Results

Overall, the weighted prevalence of falls in the last two years was 28.4%. Prevalence of falls was higher in women (29.1%) than in men (23.5%).

Table 1 shows the prevalence and crude odds ratios (ORs) for falls in men and women according to sociodemographic and lifestyle factors. In both sexes, risk of falls increased with age. There were no associations in either sex between risk of falls and either marital status or household wealth. Relationships between lifestyle factors and falls varied by sex. In men, risk of falls was higher in ex-smokers, in those who were sedentary, and was reduced in those who drank alcohol compared to those who did not. There was no association in men between BMI and risk of falls. In women, risk of falls was higher in those who were obese, but there were no associations between fall risk and any other lifestyle factor.

**Table 1.** Prevalence and crude odds ratios for falls in the last two years by sociodemographic and lifestyle factors in men (*n* = 1994) and women (*n* = 2357) aged 60 and over

Characteristics	Men			Women		
	No.^a^	%^b^	OR (95% CI)	No.^a^	%^b^	OR (95% CI)
Age (years)						
60–69	1051	20.8	1.0	1270	26.6	1.0
70–79	695	27.7	1.36 (1.08, 1.72)	843	30.5	1.15 (0.95, 1.39)
≥80	198	33.2	1.71 (1.21, 2.41)	244	35.1	1.52 (1.11, 2.08)
Marital status						
Has partner	1526	24.1	1.0	1441	27.3	1.0
Divorced/ widowed	324	25.7	1.07 (0.80, 1.41)	822	31.6	1.19 (0.99, 1.44)
Single	94	27.0	1.33 (0.84, 2.10)	94	36.1	1.58 (1.02, 2.45)
Household wealth, quintiles						
1. (Poorest)	207	30.7	1.21 (0.83, 1.77)	322	32.6	1.23 (0.91, 1.66)
2.	311	21.5	0.81 (0.58, 1.16)	416	26.1	0.74 (0.61, 0.98)
3.	428	23.1	0.99 (0.73, 1.34)	565	28.5	0.89 (0.69, 1.15)
4.	502	24.5	0.98 (0.73, 1.31)	519	26.9	0.91 (0.69, 1.18)
5. (Richest)	496	24.5	1.0	535	31.7	1.0
BMI						
<25	442	24.9	1.0	695	25.4	1.0
25–29	971	22.1	0.92 (0.71, 1.20)	905	26.9	1.13 (0.90. 1.41)
≥30	531	28.5	1.09 (0.82, 1.47)	757	34.6	1.53 (1.22, 1.92)
Smoking status						
Never	538	21.2	1.0	1027	28.7	1.0
Ex-smoker	1224	26.2	1.33 (1.04, 1.70)	1125	30.8	1.15 (0.96, 1.39)
Current smoker	182	23.6	1.14 (0.75, 1.72)	205	22.3	0.88 (0.62, 1.23)
Physical activity						
Sedentary	59	47.2	2.57 (1.42, 4.66)	74	39.3	1.51 (0.87, 2.61)
Light	363	27.4	1.31 (0.95, 1.81)	638	31.8	1.22 (0.93, 1.60)
Moderate	1014	23.2	1.16 (0.89, 1.50)	1246	26.8	0.83 (0.64, 1.06)
Vigorous	508	21.1	1.0	399	28.6	1.0
Frequency of alcohol intake						
Almost every day	398	25.9	0.70 (0.47, 1.04)	272	31.1	1.03 (0.73, 1.45)
Once or twice a week or more often	973	22.6	0.61 (0.42, 0.88)	950	26.7	0.80 (0.61, 1.05)
Once or twice a month	223	25.3	0.76 (0.49, 1.18)	284	30.5	1.02 (0.73, 1.43)
Once or twice a year or more often	186	22.1	0.45 (0.27, 0.75)	495	30.1	1.00 (0.75, 1.36)
Not at all	164	33.0	1.0	356	30.5	1.0

^a^Unweighted bases.

^b^Weighted prevalence of falls.

Table 2 shows the prevalence and crude ORs for falls in men and women according to medical factors and physical and cognitive functions. Looking first at medical factors, in both men and women, risk of falls was higher in those who were taking more prescribed medications, in those with greater co-morbidity, in those who were frail or pre-frail, in those who reported problems with incontinence, in those who were troubled by moderate or severe pain and in those who had a high level of depressive symptoms. In men, risk of falls was greater only in those who reported poor eyesight and tended to be greater in those who reported poor hearing, although this latter association was of borderline significance. As regards physical function, in both sexes risk of falls was greater in those with the poorest grip strength and in those who either had the slowest walking speed or did not attempt the walking test for safety or health reasons. In both sexes, risk of falls was greater in those who did not attempt the full-tandem balance stand. Men and women who were unable to keep their balance in this test for 10 s also had a higher risk of falls but this was only statistically significant in men. In men, but not in women, those with the poorest forced expiratory volume in one second (FEV1) had a higher risk of falls. There was no association in either sex between memory performance and likelihood of falling.

**Table 2.** Prevalence and crude odds ratios for falls in the last two years by medical factors and physical and cognitive functions in men and women aged 60 and over

Characteristics	Men			Women		
	No.^a^	%^b^	OR (95% CI)	No.^a^	%^b^	OR (95% CI)
No. of medications						
≤1	643	17.9	1.0	815	22.4	1.0
2–4	675	22.7	1.20 (0.92, 1.57)	851	30.5	1.54 (1.24, 1.92)
≥5	625	32.8	1.93 (1.48, 2.52)	691	34.2	1.65 (1.31, 2.08)
No. of diagnosed co-morbid conditions^c^						
0	775	17.4	1.0	851	22.1	1.0
1	953	27.0	1.38 (1.11, 1.72)	701	29.3	1.58 (1.24, 2.01)
2	480	29.1	1.90 (1.48, 2.44)	276	36.2	1.85 (1.35, 2.52)
≥3	149	33.2	2.07 (1.43, 3.01)	116	38.7	2.11 (1.38, 3.23)
Frailty status						
Not frail	1035	18.9	1.0	1187	23.9	1.0
Pre-frail	691	23.7	1.33 (1.05, 1.68)	864	30.1	1.39 (1.14, 1.69)
Frail	160	46.8	3.75 (2.61, 5.40)	243	48.0	3.10 (2.30, 4.17)
Poor eyesight						
No	1771	22.8	1.0	2093	29.5	1.0
Yes	173	40.3	1.94 (1.42, 2.74)	264	26.4	0.93 (0.70, 1.24)
Poor hearing						
No	1378	23.0	1.0	1971	28.4	1.0
Yes	566	28.3	1.22 (0.98, 1.53)	386	32.4	1.13 (0.89, 1.43)
Incontinence						
No	1768	23.3	1.0	1823	26.8	1.0
Yes	176	37.2	1.50 (1.07, 1.10)	534	37.0	1.71 (1.31, 2.09)
Troubled by pain						
No	1238	19.4	1.0	1289	24.2	1.0
Mild pain	248	23.6	1.30 (0.94, 1.79)	305	30.1	1.30 (0.98, 1.71)
Moderate pain	340	30.9	1.73 (1.32, 2.27)	587	32.1	1.50 (1.21, 1.86)
Severe pain	118	41.1	2.75 (1.85, 4.10)	176	50.2	3.01 (2.18, 4.17)
Depressive symptoms (CES-D)						
≥3	1239	29.8	1.53 (1.28, 1.83)	1136	33.5	1.64 (1.32, 2.03)
<3	705	19.6	1.0	1221	24.0	1.0
Balance (full-tandem stand)						
10 s	1622	21.1	1.0	1811	27.0	1.0
<10 s	226	29.5	1.53 (1.12, 2.09)	380	31.6	1.23 (0.96, 1.56)
Not attempted	96	54.3	3.91 (2.57, 5.97)	166	40.1	2.27 (1.64, 3.16)
Walking speed, quartiles						
1 (Lowest^d^)	385	30.0	1.62 (1.18, 2.21)	589	38.6	1.96 (1.51, 2.53)
2	566	25.4	1.29 (0.97, 1.72)	560	26.6	1.17 (0.90, 1.52)
3	481	18.3	0.88 (0.64, 1.20)	601	26.2	1.13 (0.87, 1.46)
4 (Highest)	505	20.7	1.0	599	23.9	1.0
Grip strength, quartiles						
1 (Lowest)	459	33.2	2.02 (1.51, 2.72)	554	36.1	1.46 (1.15, 1.86)
2	472	21.7	1.19 (0.87, 1.62)	603	28.8	1.05 (0.82, 1.33)
3	491	21.5	1.22 (0.90, 1.65)	482	23.5	0.82 (0.63, 1.07)
4 (Highest)	522	16.8	1.0	718	27.0	1.00
Lung function (FEV)						
1 (Lowest)	552	28.4	1.37 (1.00, 1.88)	654	32.9	1.22 (0.93, 1.61)
2	544	23.3	1.09 (0.79, 1.50)	698	29.4	1.06 (0.81, 1.40)
3	483	20.2	0.95 (0.68, 1.33)	605	24.6	0.84 (0.63, 1.12)
4 (Highest)	385	20.1	1.0	400	28.0	1.0
Memory, quartiles						
1 (Lowest)	336	26.0	0.94 (0.69, 1.27)	436	31.7	0.97 (0.73, 1.29)
2	627	24.2	1.06 (0.73, 1.54)	492	30.1	0.94 (0.72, 1.24)
3	251	27.2	0.76 (0.56, 1.03)	570	30.1	0.77 (0.59, 1.00)
4 (Highest)	730	20.1	1.0	859	25.6	1.0

^a^Unweighted bases.

^b^Weighted prevalence of falls.

^c^Based on diagnoses of heart attack, heart failure, stroke, chronic lung disease, diabetes, arthritis, Parkinson's, disease, dementia, psychiatric illness, cancer and osteoporosis.

^d^Includes those unable to do the walking speed test.

We carried out multivariable analysis in men and women separately including all factors shown in Tables 1 and 2 that were associated with a history of falling with a *P* value of <0.2. Table 3 shows the factors that were independently associated with risk of falls in men and women respectively after multivariable analysis. In men, significant independent risk factors for a history of falling were severe pain, high levels of depressive symptoms, being unable to attempt the balance test and having been diagnosed with a chronic disorder. Men diagnosed with two or more chronic disorders also were slightly more likely to have a history of falling but these associations were not significant. Older age was associated with a slight increase in risk. In women, severe pain and having two or more chronic disorders were also significant independent risk factors for a history of falling. Sex-specific risk factors in women were incontinence and being frail. In contrast to men, increasing age, depressive symptoms and being unable to attempt the balance test were not independent correlates of falling in women.

**Table 3.** Independent risk factors for falls in men and women aged 60 and over

Characteristics	Adjusted^a^ OR (95% CI)
Men	
Troubled by pain	
No	1.0
Mild	1.23 (0.89, 1.71)
Moderate	1.32 (0.98, 1.77)
Severe	1.92 (1.26, 1.94)
No. of diagnosed co-morbid conditions	
0	1.0
1	1.40 (1.08, 1.81)
2	1.38 (0.98, 1.94)
≥3	1.13 (0.69, 1.85)
Depressive symptoms (CES-D)	
≥3	1.33 (1.05, 1.68)
<3	1.0
Balance (full-tandem stand)	
10 s	1.0
<10 s	1.27 (0.91, 1.78)
Not attempted	3.32 (2.09, 5.29)
Age, yrs	1.02 (1.07, 1.04)
Women	
Troubled by pain	
No	1.0
Mild pain	1.15 (0.86, 1.54)
Moderate pain	1.20 (0.95, 1.53)
Severe pain	1.90 (1.32, 2.74)
Incontinence	
No	1.0
Yes	1.48 (1.19, 1.85)
Frailty status	
Not frail	1.0
Pre-frail	1.07 (0.87, 1.37)
Frail	1.69 (1.06, 2.69)
No. of diagnosed co-morbid conditions	
0	1.0
1	1.14 (0.90, 1.45)
2	1.33 (1.00, 1.78)
≥3	1.22 (0.79, 1.89)

^a^In men, multivariable model contained age (as a continuous variable), smoking status, physical activity, frequency of alcohol intake, number of medications, number of diagnosed co-morbid conditions, frailty status, troubled by pain, poor eyesight, incontinence, depressive symptoms, balance, walking speed, grip strength and lung function. In women, multivariable model contained age (as a continuous variable), BMI, number of medications, number of diagnosed co-morbid conditions, frailty status, incontinence, troubled by pain, depressive symptoms, balance, walking speed and grip strength.

## Discussion

In this large sample of men and women aged ≥60, we confirmed previous observations that women are more likely to fall than men [8, 9, 13]. After investigation of a wide range of potential risk factors—sociodemographic, lifestyle or behavioural, medical, and physical and cognitive functions—we found that higher levels of pain and the presence of chronic disorders were independently associated in multivariable analysis with an increased likelihood of having a history of falls in both men and women. There were also some sex-specific risk factors. In men, increased likelihood of falls was associated with high levels of depressive symptoms, older age and being unable to take the balance test, while in women likelihood of falls was associated with urinary incontinence and frailty.

Our observation of a relationship between level of pain and history of falls in both sexes is consistent with recent findings in the MrOS (Osteoporotic Fractures in Men) cohort of men aged 65 and over where likelihood of subsequent falls was increased in those reporting that pain had a moderate or severe effect on their normal activities at baseline [24]. We found evidence of a dose–response relation between level of pain and falls in both men and women, though likelihood of falls was only significantly increased in men reporting severe pain and in women reporting either moderate or severe pain. This finding adds to previous evidence on the importance of pain intensity for fall risk [25]. We were not able to examine whether pain at specific sites was linked to falls.

Previous evidence indicates that deficits in balance and in muscle function are risk factors for falls [15–17]. Here, we found that in men, only being unable to attempt the full-tandem stand balance test was associated with increased likelihood of falls but shorter full-tandem stance time was not; this might be because men considered at risk of falling were discouraged from attempting this test. Being unable to attempt the full-tandem stand balance test was also associated with likelihood of falls in women in multivariable analysis, but this became non-significant once frailty status was included in the model (data not shown). Poor grip strength was not associated with fall risk in multivariable analysis. This is consistent with observations in the very large Prospective Urban-Rural Epidemiology study of people aged 35–70 years where grip strength was not predictive of hospital admission due to a fall [26], though evidence from a meta-analysis of longitudinal studies in people aged 65 or over found that muscle weakness, and especially lower extremity weakness, significantly increased the risk of falls [17]. We had no measure of lower extremity muscle strength.

A recent systematic review demonstrated that frailty increases the risk of future falls in community-dwelling older people, and that this risk seems to be higher in men [18]. In the current cross-sectional analysis, frailty was associated with a history of falls in both sexes in univariate analysis, but after adjustment for other risk factors, it was only significantly associated with falls in women.

There is evidence from many studies that depressive symptoms are associated with a higher likelihood of falls [19]. Our findings in men in the current study are consistent with that. However, in women the association between greater depression and falls ceased to be significant in multivariable analysis once frailty was included in the model (data not shown), suggesting that the association—in this sex at least—may be explained by common pathways such as slow walking speed, weakness and exhaustion.

Urinary incontinence is a recognised risk factor for falls [20]. In the current study, the association was present in both sexes in unadjusted analyses but only persisted in women after multivariable analyses, perhaps because the prevalence of incontinence was markedly higher in women (23% versus 9%).

In contrast to findings in several other studies [13, 15], we found that the dose–response association seen in both sexes between greater number of co-morbid conditions and increased likelihood of falls in unadjusted analyses was markedly attenuated after adjustment for a range of other risk factors in multivariable analysis. In men, the dose–response association disappeared and an increased risk of falls was evident only in those with at least one disorder. In the current study, we were able to adjust for a number of factors that could potentially have confounded the association between the number of co-morbid conditions and the likelihood of falls, including physical function, depressive symptoms and pain. In studies that reported associations between extent of co-morbidity and likelihood of falls, no adjustment was made for these factors [15, 21, 22].

The strengths of our study include the large sample size, the fact that it is representative of the community-dwelling English population aged 60 and over and the availability of data on a wide range of potential risk factors for falls. It also has some weaknesses. Firstly, the cross-sectional nature of this prevalence study makes it impossible to be certain of the direction of effect in the case of some of the potential risk factors for falls that we considered such as depressive symptoms. Secondly, our analysis was based on those who completed both the initial interview for the Wave 6 survey and agreed to be visited at home by a nurse for measurements of physical function. This represented 73% of those who took part in the initial interview. Data were weighted to correct for non-response bias. Finally, there was no definition in the questionnaire of what constituted a fall. Studies have varied in how a fall is defined and this diversity complicates comparison of findings [27].

In this cross-sectional survey of a nationally representative sample of men and women aged ≥60, we confirmed previous observations that risk of falls is greater in women and provided further evidence that the aetiology of falls is multifactorial. Although we found some homogeneity between the sexes in that higher levels of pain and the presence of chronic disorders were associated with increased likelihood of falls in both men and women, some risk factors were sex-specific, namely incontinence and frailty in women, and depressive symptoms and poor balance in men. Design of fall-prevention strategies should take gender into account.

Key points

- Falls are a major cause of death and disability in older people.
- Falls occur more commonly in women but it is unclear whether risk factors for falls vary between the sexes.
- Severe pain and chronic disease were associated with increased likelihood of falls in both men and women.
- Several risk factors were sex-specific, namely incontinence and frailty in women, and depression, older age, and poor balance in men.

## Supplementary data

Supplementary data mentioned in the text are available to subscribers in *Age and Ageing* online.

Supplementary Data

Supplementary Data
