# Supplementary material for: Prevalence and risk factors for falls in older men and women: The English Longitudinal Study of Ageing
Source: Age Ageing. 2016 Nov 2;45(6):789–94. doi: 10.1093/ageing/afw129 (PMC5105823; doi:10.1093/ageing/afw129)
Supplement: Supplementary Data [file supp_45_6_789__index.html]

Prevalence and risk factors for falls in older men and women: The English Longitudinal Study of Ageing — Prevalence and risk factors for falls in older men and women: The English Longitudinal Study of Ageing — Supplementary Data 

# Prevalence and risk factors for falls in older men and women: The English Longitudinal Study of Ageing

## Supplementary Data

Supplementary Data

- Supplementary Data - docx file
