# Supplementary material for: Prevalence and risk factors for falls in older men and women: The English Longitudinal Study of Ageing
Source: Age Ageing. 2016 Nov 2;45(6):789–94. doi: 10.1093/ageing/afw129 (PMC5105823; doi:10.1093/ageing/afw129)
Supplement: Supplementary Data [file supp_afw129_Supplementary_file_prevalence_and_risk_factors_for_falls.docx]

**Supplementary file**

**Details of assessment of independent variables**

BMI was derived from weight and height measured during a home visit by a nurse and classified as underweight/normal weight (<25 kg/m^2^), overweight (25.0-29.9 kg/m^2^) or obese (≥30 kg/m^2^). Smoking status was defined as current smoker, ex-smoker or never smoked. Participants were asked about the level of physical activity involved in their job (if in work) and responded to three questions on mild, moderate or vigorous physical activity carried out in daily life. The answers to these questions were used to derive a categorical summary variable on physical activity (sedentary, low, moderate or high) that approximates as closely as possible to the classification used in the Allied Dunbar Survey of Fitness.[1] Frequency of alcohol intake in the last 12 months was categorized into five categories, ranging from ‘almost every day’ to ‘not at all.’

Details of medications being taken were recorded by a nurse and were grouped into three categories (≤1, 2-4, ≥5). Participants were asked whether they had ever been told by a doctor that they had any of the following: hypertension or high blood pressure, angina, heart attack or myocardial infarction, congestive heart failure, diabetes, stroke, chronic lung disease, asthma, arthritis, Parkinson’s disease, Alzheimer’s disease or dementia, psychiatric or emotional problems, cancer or osteoporosis. We summed the number of conditions present as an indicator of extent of comorbidity. Depressive symptoms were assessed with the 8-item version of the Centre for Epidemiologic Studies Depression Scale (CES-D).[2] Frailty or pre-frailty according to the Fried phenotype is defined as the presence of 3 or more, or 1 or 2 respectively, of the following components: unintentional weight loss, weakness, self-reported exhaustion, slow walking speed and low physical activity.[3] We operationalized these criteria using definitions similar to those used in the original phenotype of frailty studies:[3, 4] weight loss was defined as *either* loss of ≥10% of body weight since Wave 5 *or* current BMI <18.5 kg/m^2^; weakness was defined as maximum grip strength in the lowest 20% of the distribution, after taking sex and BMI into account; exhaustion was considered present if the participant gave a positive response to either of the CES-D questions ‘Felt that everything I did was an effort in the last week’ or ‘Could not get going in the last week’; slow walking speed was defined as a walking speed in the lowest 20% of the distribution, after taking account of sex and height; and low physical activity was defined as physical activity in the lowest sex-specific 20% of the distribution. Participants were asked whether in the last 12 months they had lost any amount of urine beyond their control. Pain was assessed by asking participants if they were often troubled by pain. Those who answered positively were asked to rate the pain as mild, moderate or severe. Participants were asked to rate their hearing (with a hearing aid if used) using four categories as excellent, good, fair or poor. We grouped these responses into two categories: excellent/good/fair versus poor. Participants were asked to rate their eyesight (with glasses if used) using five categories as excellent, very good, fair, poor or registered or legally blind. We grouped these responses into two categories: excellent/very good/fair versus poor or blind.

Balance, grip strength and lung function were measured by a nurse using standardized protocols during a home visit. All participants for whom it was judged safe or who felt it would be safe to do the procedure were asked to try to stand with their feet together, side by side for about 10 seconds. The nurse recorded whether or not they were able to hold the position for 10 seconds. Grip strength was measured three times using each hand using a Gripometer. The maximum of these measurements was used in the analysis. Lung function was measured using a NDD Easy On Spirometer. The highest technically satisfactory measure of forced expiratory volume in one minute (FEV1) was used in the analysis. Walking speed was assessed in participants by measuring the time taken to walk a distance of 8 feet at usual pace. Before participants performed the test, the interviewer assessed whether they could do so safely. Use of walking aids were permitted. Participants who had to rely on the support of another person or who were assessed as being in danger of falling were not asked to take the test. The timed walk was repeated and the mean of the two measurements was calculated. Immediate and delayed verbal memory was assessed by presenting a list of 10 nouns aurally on a computer, one every two seconds. Participants were asked to recall as many words as possible immediately and again after a short delay. Total combined score on these two tests was used in the analysis.

**References**

1. Activity and Health Research. Allied Dunbar National Fitness Survey: main findings. London: 1992.

2. Steffick DE, The HRS working group. Documentation of affective functioning measures in the Health and Retirement Study. HRS/AHEAD Documentation Report DR-005 [online report]. 2000 [updated 2000]; Available from: <http://hrsonline.isr.umich.edu/sitedocs/userg/dr-005.pdf>.

3. Fried LP, Tangen CM, Walston J, Newman AB, Hirsch C, Gottdiener J, et al. Frailty in older adults: evidence for a phenotype. J Gerontol A Biol Sci Med Sci. 2001;56:M146-M56.

4. Bandeen-Roche K, Xue QL, Ferrucci L, Walston J, Guralnik JM, Chaves P, et al. Phenotype of frailty: characterization in the women's health and aging studies. JGerontolA BiolSciMedSci. 2006;61:262-6.
